# SERS-imprinted nanosensors for monitoring of pesticides up to femtomolar concentrations: fusing selectivity with sensitivity

**DOI:** 10.1039/d6ra03017c

**Published:** 2026-07-02

**Authors:** Nazia Tarannum, Deepak Kumar, Pallavi Chaudhary, Pierre Dramou, Raj Kishore Sharma, Shobhita Singal

**Affiliations:** a Department of Chemistry, Chaudhary Charan Singh University Meerut 250004 India naz1012@gmail.com deepakkumar7669@gmail.com pallavi89khatri@gmail.com pierred@cpu.edu.cn; b Department of Analytical Chemistry, China Pharmaceutical University Nanjing 210009 China; c Department of Chemistry, University of Delhi 110007 India drrajksharma@yahoo.co.in; d Department of Chemistry, Motilal Nehru College, University of Delhi 110021 India shobhitasingal@gmail.com

## Abstract

Pesticide residues in food and environmental matrices pose serious health and ecological risks, demanding rapid, sensitive, and on-site detection methods. Surface-enhanced Raman spectroscopy (SERS) offers exceptional sensitivity but often lacks selectivity in complex samples. This limitation is overcome by integrating SERS with molecularly imprinted polymers (MIPs), creating SERS-imprinted nanosensors that combine molecular recognition with signal amplification. This review systematically examines the design, fabrication, and application of MIP-SERS nanosensors for pesticide detection from 2010–2025. We discuss advanced imprinting strategies (including dummy templates, stimuli-responsive systems, and controlled polymerization) and highlight the role of nanomaterials in enhancing SERS performance. These sensors achieve detection limits as low as femtomolar concentrations for pesticides such as thiram, fenthion, and carbendazim in food, soil, and water samples. We also address key challenges in reproducibility, selectivity, and field deployment, and outline future directions including portable platforms, multiplex detection, and emerging techniques like TERS and UV-SERS. This work provides a roadmap for developing robust, selective, and field-deployable nanosensors for sustainable agriculture and food safety.

## Abstract 2

Pesticide residues in food and environmental matrices pose serious health and ecological risks, demanding rapid, sensitive, and on-site detection methods.

## Graphical abstract

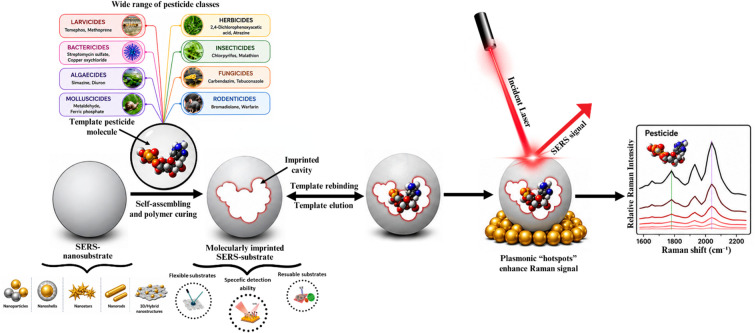

## 1. Introduction

**Fig. 1:**
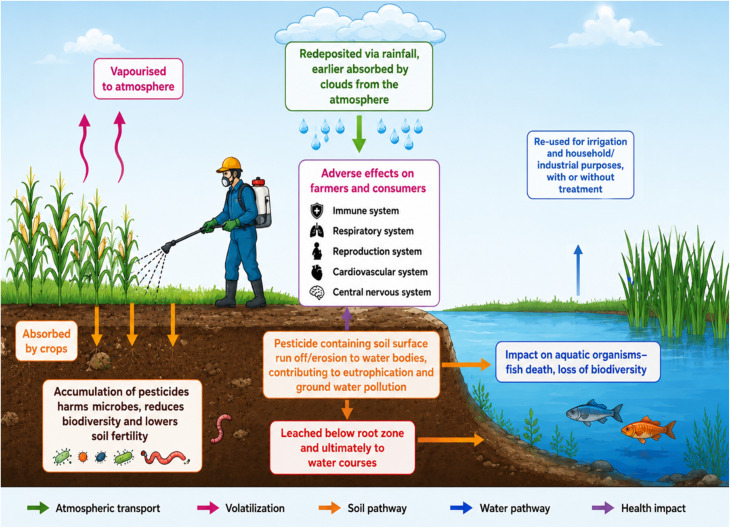
Routes of pesticide pollution and how they affect ecosystems, human health, soil, and water.

Pesticide detection is a growing field of research due to its direct implications on food safety, environmental sustainability and public health. Agriculture remains the backbone of many economies, especially in India. It contributes about 16% to the GDP of the country and supports over 46% of the population as of 2024.^1^ However, pest-related crop losses and the widespread use of chemical pesticides to mitigate them pose significant health and ecological risks. These pesticide residues can cause serious health issues including endocrine disruption, neurotoxicity and even cancer. They may accumulate within the food chain and harm non-target organisms such as pollinators and aquatic life. In addition to these biological effects, environmental pollution is another pressing concern. Runoff from agricultural fields introduces harmful chemicals into water bodies, contributing to eutrophication and groundwater pollution. In soil, the accumulation of these pesticides can harm beneficial microbes as shown in (Fig. 1). This reduces biodiversity and depletes soil fertility over time.^2–4^ Given these serious concerns, effective monitoring of pesticide residues has become essential to ensure food safety and environmental sustainability. However, detecting pesticides in food and environmental samples is a major analytical challenge because they are present in very small amounts and the samples are often complex. Conventional analytical methods such as gas chromatography-mass spectrometry (GC-MS), liquid chromatography-mass spectrometry (LC-MS), high-performance liquid chromatography-mass spectrometry (HPLC-MS) and immunoassay are widely used due to their high sensitivity and selectivity.^3–6^ Several recent studies, such as those by Meng *et al.* (2021), Oprita *et al.* (2022), Tsagkaris *et al.* (2022) and Xu *et al.* (2022) demonstrated the effective application of these techniques for identifying different pesticide residues in food samples, achieving detection limits (LODs) as low as micrograms per kilogram (µg kg^−1^).^7–11^ Despite their analytical strengths, these chromatographic techniques have significant drawbacks. First, they involve sample preparation such as extraction, purification and derivatization steps that are both time-consuming and require considerable manual effort. Second, the instruments used are expensive and need trained personnel to operate them properly. Third, they contribute to environmental pollution by using an excessive amount of organic solvents. Sample pretreatment is another drawback, which increases analysis time and complexity. In addition, these systems are not portable and cannot be used for on-site and large-scale screening, which limits their practical utility in field conditions.

These challenges highlight the need for alternative detection systems that are simple, cost-effective and capable of delivering fast results without extensive sample processing. Such systems should be portable, user-friendly and environmentally sustainable. To address the limitations of conventional detection methods, surface-enhanced Raman spectroscopy (SERS)-based nanosensors integrated with molecularly imprinted polymers (MIPs) have emerged as a promising solution.^12^

SERS provides high sensitivity and selectivity by amplifying weak Raman signals through the interaction of analyte molecules with metallic nanostructures. The use of gold (Au) and silver (Ag) nanoparticles, hybrid nanocomposites, carbon nanotubes, carbon dots and silica-based materials as SERS substrates allows detection at very low concentrations.^13^ However, conventional SERS suffers from low selectivity, particularly when structurally similar molecules produce overlapping Raman spectra. This makes it difficult to accurately identify the target pesticide molecule from other in complex samples. To overcome this, researchers have integrated MIPs with SERS-active nanomaterials, creating SERS-based imprinted nanosensors.^14^

MIPs are synthetic materials that designed to recognize specific molecules in a manner similar to the way antibodies recognize antigens. They are formed by polymerization of monomers and cross-linkers around a template molecule, which can be a small molecule, protein or cell. After polymerization, the template is washed off which results in the formation of well-defined molecular cavities that match the shape, size and functional group orientation of the original analyte.^15^ The design of these cavities allows them to recognize and bind specifically to the target, functioning in a manner similar to a lock-and-key mechanism. MIPs are often preferred over natural receptors because they offer better stability and can be manufactured at lower production costs.^16–20^ Their integration with SERS enables simultaneous achievement of molecular selectivity and high sensitivity, even in complex matrices like as food, water and soil. This hybrid approach has revolutionized pesticide detection, making SERS based imprinted nanosensors a powerful tool for ensuring food and environmental safety.^21^ SERS-based imprinted nanosensors merge high sensitivity from the local field enhancement of optically resonant metal nanoparticles with comprehensive chemical fingerprint data obtained *via* Raman spectroscopy. This causes Raman signals to be strongly amplified and makes it possible to identify molecular species with great sensitivity without the need for labels. SERS-based imprinted nanosensor testing can be carried out in ambient circumstances in an aqueous solution. It is hence appropriate for use in analytical chemistry. Significant progress has been made in this field, from studies using roughened metallic surfaces to advanced nanoscale platforms for single-molecule detection, sensing and imaging applications.^22,23^

Although numerous reviews have addressed SERS-based sensing platforms, molecularly imprinted polymers, and nanomaterial-assisted sensing platforms separately, a critical assessment of how molecular imprinting influences the analytical performance of SERS systems remains limited. In particular, important factors such as analyte positioning relative to electromagnetic hotspots, the effect of MIP thickness and architecture on signal enhancement, trade-offs between selectivity and sensitivity, and challenges associated with reproducibility and practical deployment have not been comprehensively evaluated. To address these gaps, the present review examines MIP-SERS platforms from a design and performance perspective. Rather than solely summarizing reported detection results, this review compares different MIP-SERS configurations used for pesticide detection, highlighting how variations in structural design influence recognition efficiency, hotspot accessibility, signal amplification, sensitivity, selectivity and reproducibility. Furthermore, emphasis is placed on identifying the factors responsible for ultralow detection limits. By integrating literature reported between 2010 and 2025, discussing current limitations and highlighting emerging research directions, this review provides practical design insights for the development of next-generation MIP-SERS nanosensors for food safety and environmental monitoring.

## 2. Background and importance of pesticide detection

**Fig. 2:**
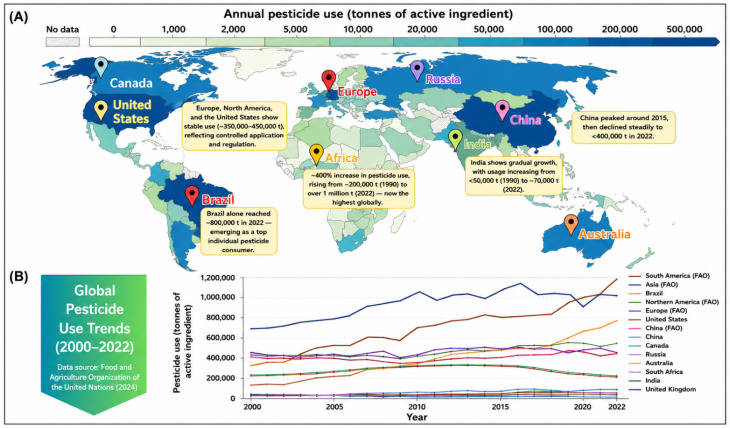
Global pesticide use and regional trends. (A) Global distribution of annual pesticide use (tonnes of active ingredient). Color indicates total use based on the scale shown at the top and callout boxes highlighting key regional trends. (B) Line graph showing temporal trends in pesticide use (tonnes of active ingredient) across major countries and regions from 2000 to 2022.

The rise in global food demand has led to intensified agricultural practices and increased pesticide application to ensure higher crop yields. The consumption of chemical pesticides has shown yearly and regional variations depending on factors like the area covered under cultivation, agro climatic conditions, cropping intensity, soil quality and the presence of pests and diseases.^24^ According to Ritchie *et al.*, (2023), global pesticide consumption has steadily increased over the past decades, exceeding 3.7 million metric tonnes of active ingredients in recent years. The highest pesticide usage is concentrated in regions with large-scale commercial farming, including Asia, the Americas and Europe, with China, Brazil and the United States being among the top consumers as shown in (Fig. 2).^25^ Against this global backdrop, India also demonstrates significant pesticide consumption trends. According to the most recent figures from the Government of India (2021–2024), pesticide consumption varies significantly between states, with Uttar Pradesh ranks highest at 11 828 MT, followed by Maharashtra at 8718 MT and Punjab with 5270 MT.^26^

This high consumption reflects the need for pest control but also raises concerns over pesticide traces in food and the environment. Many commonly used pesticides like organophosphates, carbamates, neonicotinoids and pyrethroids have been linked to severe health and environmental effects. These include hormonal disruption, neurological damage and potential carcinogenicity. In ecosystems, bioaccumulation affects pollinators, aquatic life and overall biodiversity. Pesticide runoff contributes to water pollution and soil degradation by affecting microbial balance and fertility.^2–4^ To address these concerns, national and international regulatory agencies like the Food Safety and Standards Authority of India (FSSAI), the Food and Agriculture Organization (FAO), the World Health Organization (WHO) and the European Food Safety Authority (EFSA) have set Maximum Residue Limits (MRLs) for pesticides in food and agricultural products (Table 1).^27^ These MRLs define the highest level of pesticide residue that is legally permitted to remain in or on food products. This regulatory framework highlights the urgent need for rapid, sensitive and portable methods to detect pesticide residues in food, soil and water.

**Table 1:** Classification of pesticides based on LD_50_ values in rats

Class	Hazardous level	LD_50_ for the rats (mg per kg body weight)		Example
		Dermal	Oral	
Ia	Extremely hazardous	<50	<5	Parathion, aldicarb, methyl parathion
Ib	Highly hazardous	50–200	5–50	Carbofuran, monocrotophos, methomyl
II	Moderately hazardous	200–2000	50–2000	Chlorpyrifos, cypermethrin, deltamethrin
III	Slightly hazardous	>2000	>2000	Imidacloprid, malathion, acetamiprid
IV	Unlikely to present acute hazard	>5000	>5000	Azadirachtin, *Bacillus thuringiensis*, pymetrozine

## 3. Integration of SERS and MIPs: design and working mechanism of SERS-based imprinted nanosensors

SERS-based imprinted nanosensors combine two core components: a SERS-active metallic substrate, typically composed of AuNPs or AgNPs to amplify Raman signals and a molecularly imprinted polymer layer that offers selective molecular recognition through imprinted binding sites.^12,13^ The process starts with the fabrication and surface modification of the metallic nanoparticles to form the SERS-active substrate. Subsequently, a pre-polymer complex forms between the functional monomer and the target molecule. These monomers align themselves around the template molecule by forming molecular aggregates through either covalent or non-covalent interactions. After that, cross-linkers are introduced to connect these monomers during the polymerization process. When polymerization process is complete, the template molecule is washed off which results in the formation of well-defined molecular cavities that match the target molecule in shape, size and functional group orientation. These imprinted sites allow the sensor to selectively capture the target molecule on the metal surface, where SERS can then generate a detectable signal.^28^

**Fig. 3:**
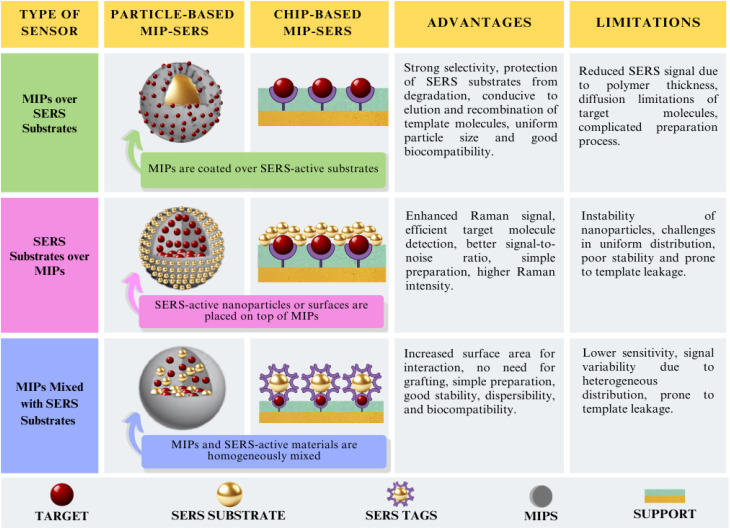
Three primary structural formats are employed in the design of MIP-SERS nanosensors: (i) MIPs applied on top of SERS substrates, (ii) SERS-active substrates positioned over MIPs and (iii) uniform mixing of SERS substrates and MIPs.

The structural design of MIP-SERS nanosensors primarily falls into two categories: particle-based and chip-based MIP-SERS nanosensors. Particle-based nanosensors use metal nanoparticles like gold or silver as the core material. These particles provide a large surface area and generate strong electromagnetic fields, enhancing Raman signals. This format works well in liquid samples where nanoparticles can interact freely with analytes. For example, Sitjar *et al.* (2020) published an MIP nanosensor based on gold nanoparticles that detected the pesticide, cypermethrin, at sub-nanomolar concentrations.^29^ In contrast, chip-based nanosensors are more suited for on-site testing. These use solid platforms with uniformly distributed plasmonic sites and MIP layers to enable consistent and reproducible detection. Mu *et al.* (2019) developed a smartphone-based chip-integrated Raman sensor for multiplex detection of pesticide residues with minimal interference.^30^ Each structural design presents unique advantages and limitations regarding sensitivity, reproducibility, stability and fabrication complexity. The following (Fig. 3) provides a structural classification of these MIP-SERS nanosensors and outlines their advantages along with their limitations.

As shown in the Fig. 3, the performance of MIP-SERS nanosensors in terms of efficiency and accuracy is influenced by the molecular imprinting process. The formation of recognition sites plays a key role in determining how effectively the sensor identifies and binds to target pesticide molecules, particularly in complex food and environmental samples. The selection of an appropriate imprinting strategy is also guided by factors such as the chemical stability of the template and the complexity of the sample matrix.^31^ As a result, researchers have developed several imprinting approaches-each tailored to address specific challenges such as improving selectivity, avoiding template leakage, enabling reusability or enhancing binding affinity.^32^ The following sections explore imprinting strategies that have advanced the design and performance of MIP-SERS nanosensors.

## 4. Advanced imprinting strategies of MIP-SERS nanosensors for pesticide residue detection

**Fig. 4:**
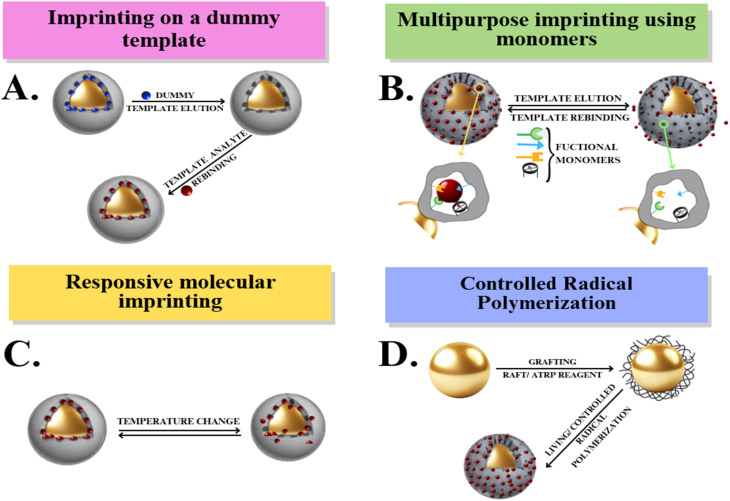
Techniques for MIP-SERS nanosensor imprinting: (A) imprinting on a dummy template, (B) multipurpose imprinting using monomers, (C) responsive molecular imprinting and (D) controlled radical polymerization.

The fabrication of MIP-SERS nanosensors for pesticide detection requires precise engineering to achieve both high molecularity, selectivity and optimized SERS signal enhancement. To address the needs for detecting trace amounts of pesticide residues in complex samples, various imprinting strategies have been developed.^18,28^ These approaches aim to improve imprinting efficiency, avoid template leakage, enabling reusability, control polymer thickness and enhance the plasmonic interactions for better sensitivity. A visual representation of different imprinting strategies employed in MIP-SERS nanosensors is presented in (Fig. 4). The figure has four parts, showing the key working principles of each method: (A) dummy template imprinting, (B) multifunctional monomer-based imprinting, (C) stimuli-responsive molecular imprinting, (D) controlled radical polymerization. Each method uses a different approach to add functional groups, select template molecules and respond to external changes.^33–41^ These differences affect how well the nanosensors can selectively recognize and detect pesticide molecules in real-world agricultural and environmental samples.

### 4.1. Dummy template imprinting for safe and selective recognition

In cases, where real target molecules are expensive, unstable or difficult to obtain, structurally similar “dummy” templates are used. These templates mimic the molecular structure of the target to create imprinted cavities. The resulting polymer cavities retain high specificity and recognition ability without requiring the real target analyte.^33^ Carboni *et al.* (2018) developed a MIP-SERS nanosensor using diethyl(4-nitrobenzyl)phosphonate as a dummy template, which is a non-hydrolyzable analog of the pesticide *p*-oxophosphorus.^34^ This design improves the performance of the nanosensor by combining the spatial selectivity of MIPs with the molecular fingerprinting ability of SERS. This method helps prevent issues such as analyte degradation or irreversible binding. This method is especially useful for detecting hazardous or toxic substances because it eliminates direct exposure to the actual analyte while still maintaining the desired molecular recognition characteristics.^35^

### 4.2. Multifunctional monomer-based imprinting for enhanced binding site specificity

One commonly employed approach for the construction of MIP-SERS nanosensors is the use of multifunctional monomers during the imprinting process. This multifunctionality improves the imprinting efficiency and the overall performance of the nanosensor, particularly for complex targets like pesticide residues. Selecting monomers with different functional groups helps in forming more stable and highly specific binding sites.^36^ A notable study by Niu *et al.* (2024) highlights recent advances in selecting suitable functional monomers for molecularly imprinted polymers, which are essential for enhancing selectivity and sensitivity in sensing applications.^37^ This approach works well in complex samples where other substances may interfere with accurate detection.

### 4.3. Stimuli-responsive MIP-SERS systems for dynamic pesticide detection

Another emerging strategy in MIP-SERS nanosensors involves materials that respond to external stimuli. These materials are designed to undergo structural or chemical changes in response to external factors like as pH, temperature or light. This enables controlled recognition and release of target molecules. This adaptability not only enhances nanosensor reusability but also allows for real-time monitoring of biological or environmental variations. For example, Kou *et al.* (2020) developed a magnetically responsive MIP-SERS nanosensor using Fe_3_O_4_@SiO_2_–Au@Ag nanocomposites with adjustable interparticle gaps to detect paclobutrazol residues in complex environments.^38^ By optimizing the nanogaps, the sensor achieved a LOD of 2.55 × 10^−7^ M for paclobutrazol in soil samples. Additionally, Seebunrueng K. *et al.* (2024) developed a thermo-responsive magnetic MIP for the selective extraction of the several organophosphorus pesticides including fenitrothion, diazinon, ethion, parathion-ethyl and fenthion using *N*-isopropyl acrylamide (NIPAM) as the thermo-sensitive monomer. These polymer structures swelled or contracted depending on the ambient temperature, enabling controlled uptake and release of pesticide molecules.^39^ This feature is particularly valuable for applications requiring reversible binding and controlled detection.

### 4.4. Controlled radical polymerization for uniform MIP layer formation

Achieving uniform polymerization and precise control over both the polymer structure and the thickness of the imprinted layer are critical for nanosensor performance. To meet these requirements, researchers turn to controlled radical polymerization methods that allow them to guide how a polymer chain grows, break and reform bonds in a predictable way. One class of these methods works by using a chain transfer agent that helps manage repeated bond-forming and bond-breaking from the growing chain, while another relies on a catalyst that switches the radical on and off in a controlled cycle. By maintaining an optimal polymer thickness, these techniques enhance both the selectivity and sensitivity of the nanosensor. For example, Beyazit *et al.* (2018) showed that controlled/living radical polymerization techniques are effective for preparing molecularly imprinted polymer nanomaterials.^40^

Collectively, these imprinting approaches address different challenges in MIP-SERS sensor design. Dummy-template imprinting improves safety and minimizes template leakage, whereas multifunctional monomers enhance binding-site specificity and recognition strength. Stimuli-responsive systems offer dynamic control over analyte capture and release, while controlled radical polymerization enables the formation of more uniform and accessible recognition sites. Consequently, the selection of an imprinting strategy depends on the target pesticide, sample complexity, and the desired balance between selectivity, response time, reproducibility, and fabrication complexity. While molecular imprinting governs selective analyte recognition, the performance of MIP-SERS sensors also depends critically on the nanomaterial substrate that generates and amplifies the Raman signal. Nanomaterials not only provide the plasmonic hotspots required for signal enhancement but also influence sensitivity, reproducibility, stability, and performance in complex sample matrices. Therefore, the rational design and selection of nanomaterials, together with appropriate imprinting strategies, are essential for developing highly sensitive, selective, and reliable SERS-based platforms for pesticide detection.^41^

## 5. Nanomaterial-based enhancements in SERS sensors for pesticide detection

Nanomaterials have gained significant interest because of their unique physical, chemical and structural characteristics. A key reason for this interest is their large surface area in relation to their volume. This enhances surface reactivity and supports better interaction with target molecules in sensor applications.^41^ Nanomaterials also possess excellent thermal and electrical conductivity, particularly in forms like graphene and nanowires.^42^ Their optical properties are adjustable, as seen in materials such as quantum dots, which makes them useful in devices for imaging, displays and sensors. Nanomaterials are also highly responsive to environmental changes, which further enhances their application in chemical detection.^43,44^ In the context of SERS, nanomaterials such as gold (Au) and silver (Ag) nanoparticles are widely used. These particles have strong plasmonic properties. They can greatly amplify weak Raman signals and allow detection of analytes even at very low concentrations. Their shape, size and composition directly influence how much the Raman signal is enhanced. For example, functionalized Au NPs have been used to detect pesticides like imidacloprid and acetamiprid in fruits such as apples and potatoes. DNA-aptamer-based nano-tetrahedron structures with Ag@Au NPs have also enabled the simultaneous detection of multiple pesticides through hotspot formation that boosts Raman signals.^45,46^ Bimetallic Au/Ag NPs and Au-coated urchin-like ZnO nanostructures have further demonstrated high sensitivity in complex environments like groundwater and food matrices.^47,48^ Metal–organic frameworks (MOFs) are another group of nanomaterials used in SERS platforms. Their porous structure allows them to pre-concentrate small molecules such as volatile organic compounds, thus enhancing sensitivity and selectivity.^49,50^ When MOF shells are used to encapsulate plasmonic nanoparticles, they help improve signal uniformity and stability in mixed samples.^51^ SERS substrates based on nanostructures have also been proven effective in amplifying Raman signals. Nanocomposites such as Ag/rGO and Fe_3_O_4_/Ag have enabled detection of pesticides like thiram, carbendazim and ametrine at extremely low limits.^52–54^ The shape, size and surface characteristics of these nanoparticles play a key role in determining signal quality. Furthermore, advanced structures such as Janus nanoparticles have been introduced. These particles allow dual-surface functionalization. One side holds reference molecules for signal calibration while the other detects the target, improving both accuracy and reliability.^55^ These features show that nanomaterials are critical to the success of SERS-based sensors. They not only improve signal strength but also support miniaturization, faster analysis and better selectivity. The Table 2 below presents different types of nanomaterials/nanoparticles used in SERS. Each nanomaterial/nanoparticle shows unique properties that enhance SERS signals and improve detection performance.

**Table 2:** Types of nanomaterials/nanoparticles used in pesticide sensing

S. no.	Nanomaterial/nanoparticle	Size (nm)	Pesticide (target molecules)	Class	LODs (M)	References
1	Gold nanosol	—	Carbendazim	Benzimidazole	8 × 10^−11^	56
2	Silver nanoparticles	24	Thiram	Dithiocarbamate	1.00 × 10^−10^	57
3	Silver nanoparticles	40–50	Malathion	Organophosphate	4.54 × 10^−7^	58
4	Gold nanostars	248	Paraquat	Bipyridylium herbicide	8 × 10^−9^	59
5	Gold nanorods	37.81 ± 4.83	Dithiocarbamate	Dithiocarbamate	42 × 10^−9^	60
6	Gold-coated silver nanoparticles	1.1–9.5	Thiram	Dithiocarbamate	1 × 10^−10^	61
7	Silver nanodendrites	90–150	Dimethoate	Organophosphate	8.73 × 10^−9^	62
8	Silver nanoparticles	35 ± 3	Thiram	Dithiocarbamate	1 × 10^−10^	63
9	Gold nanorods	230 ± 11	Malachite green	Triphenylmethane dye	1.00 × 10^−9^	43
10	Silver nanoparticles	30	Quinalphos, triazophos	Organophosphate	1.70 × 10^−9^	64
11	Gold nanoparticles	30	Acetamiprid	Neonicotinoid	1.76 × 10^−8^	65
12	Au nanorods	20–50	Tricyclazole	Triazole fungicide	1 × 10^−7^	66
13	Au nanorods	20–50	Carbaryl	Carbamate	1 × 10^−6^	66
14	Silver nano dendrites	630	Thiram	Dithiocarbamate	5 × 10^−8^	67
15	Gold nanoparticles	11–40	Chlorpyrifos	Organophosphate	2.85 × 10^−8^	68
16	Silver nanoparticles	7	Thiram	Dithiocarbamate	1 × 10^−9^	67
17	Silver nanodendrites	70–180	Permethrin	Pyrethroid	1.03 × 10^−8^	69
18	Gold nanoparticles	50	Ferbam	Dithiocarbamate	∼2.38 × 10^−7^	70
19	Gold nanoparticles	50–120	Methyl parathion	Organophosphate	3.80 × 10^−9^	71
20	Gold-coated silver nanoparticles	26	Thiacloprid, profenofos, oxamyl	Neonicotinoid, organophosphate, carbamate	∼2.65 × 10^−11^	72
21	Gold-coated silver nanoparticles	21.25	2,4 Dichlorophnenoxy acetic acid	Phenoxy herbicide	4.15 × 10^−5^	73
22	Silver nanoparticles	30–60	Carbaryl	Carbamate	∼10^−12^	74
23	Silver-coated gold nanorods	170	Permethrin, cypermethrin, carbaryl, phosmet	Pyrethroids, carbamate and organophosphate	1.00 × 10^−8^	75
24	Gold nanoparticles	9.1	Thiabendazole	Benzimidazole	4.47 × 10^−7^	76
25	Au@ZrO nanoparticles	30–45	Cypermethrin, permethrin, carbaryl, phosmet	Pyrethroids, carbamate and organophosphate	1.00 × 10^−6^	77
26	Au nanorods	27–95	Thiabendazole	Benzimidazole	6.96 × 10^−7^	78
27	Gold nanoparticles	100	Thiabendazole	Benzimidazole	∼4.97 × 10^−8^	79 and 80
28	Gold nanoparticles	19.5	Fonofos	Organophosphate	4.06 × 10^−5^	81
29	Silver-coated gold nanoparticles	20–30	Imidacloprid, acetamiprid, thiabendazole	Neonicotinoid, benzimidazole	5.63 × 10^−12^, 9.43 × 10^−12^, 1.10 × 10^−11^	82
30	Silver nanofinger chips	—	Chlorpyrifos, thiabendazole	Organophosphate, benzimidazole	9.99 × 10^−11^, 1.74 × 10^−10^	83

## 6. Role of SERS based imprinted nanosensors in pesticide residue detection

SERS has become an effective method for detecting pesticide residues due to its high sensitivity. In the initial phase of development, SERS-based nanosensors had limited applications because of low sensitivity and inconsistent signal output. Most of the initial systems used basic colloidal silver or gold nanoparticles as substrates. These platforms produced weak Raman signal enhancement and showed poor reproducibility. As a result, the detection limits often remained in the micromolar range (10^−5^ M). This section discusses how the use of advanced substrates has improved the selectivity, sensitivity and portability of SERS nanosensors. It also provides a comparison of their performance with conventional sensing methods.^84^

### 6.1. Application of SERS for detecting pesticide residues in food, soil and water samples

Monitoring pesticide residues in food and water is critically important for protecting public health and ensuring compliance with increasingly stringent regulatory standards. Conventional analytical approaches such as gas chromatography (GC), high-performance liquid chromatography (HPLC), and mass spectrometry (MS) provide high accuracy and sensitivity; however, these techniques generally require expensive instrumentation, extensive sample preparation, skilled personnel, and centralized laboratory facilities. As a result, they are often unsuitable for rapid, real-time, or field-based monitoring applications. In response to these limitations, surface-enhanced Raman spectroscopy (SERS) has emerged as a promising analytical tool because of its high sensitivity, rapid response, molecular specificity, and minimal sample preparation requirements.

The initial development of SERS-based pesticide detection focused on understanding the interaction mechanisms between pesticide molecules and plasmonic metal surfaces. Foundational studies by Sanchez-Cortes *et al.* (2001) investigated pesticides such as cyromazine and dithiocarbamate on silver and gold substrates, demonstrating that enhanced Raman signals could be generated through adsorption onto noble metal nanostructures.^85,86^ These early investigations established the fundamental feasibility of SERS for trace pesticide analysis and provided insight into adsorption behavior, molecular orientation, and enhancement mechanisms.

Following these pioneering studies, research efforts shifted toward the development of colloidal nanoparticle-based substrates, particularly silver and gold nanoparticles, for detecting organophosphate pesticides including omethoate, dimethoate, fonofos, and methamidophos.^87–89^ These colloidal systems offered relatively simple fabrication methods, strong enhancement effects, and low-cost detection platforms. At this stage, the primary objective was to demonstrate proof-of-concept detection with improved sensitivity compared to conventional Raman spectroscopy. Although promising results were achieved, several limitations became evident, including poor signal reproducibility, nanoparticle aggregation instability, and inconsistent hotspot formation, all of which affected quantitative reliability.

To overcome these challenges, subsequent studies increasingly focused on substrate engineering strategies aimed at improving enhancement efficiency and analytical reproducibility. Researchers developed nanostructured substrates with controlled morphologies such as nanostars, nanorods, nanopillars, core–shell structures, and hierarchical assemblies to optimize localized surface plasmon resonance (LSPR) effects and generate more uniform electromagnetic hotspots.^90^ Surface roughness engineering, interparticle gap control, and ordered nanostructure fabrication significantly improved sensitivity and signal consistency. In addition, hybrid materials incorporating graphene, metal oxides, polymers, and magnetic nanocomposites were introduced to enhance molecular adsorption, chemical stability, and selectivity toward target pesticide molecules.^91^

Tang *et al.* (2025) reported the development of a molecularly imprinted polymers-based electrochemical surface-enhanced Raman scattering (MIP-EC-SERS) substrate in their study titled “A three-in-one strategy of molecularly imprinted polymers-based electrochemical SERS for sensitive recognition of acetamiprid in vegetables.” The sensor achieved LOD of 3.2 × 10^−9^ M, with 13.6 times higher sensitivity compared to SERS.^92^ Sivaraj *et al.* (2025) in the work titled “Plasmonic as well as semiconductor nanoparticles working together to detect and detoxify thiodicarb pesticides using graphene oxide” developed a silver nanoparticle (Ag-NP) based nanocomposite for the detection of thidicarb in potato peels. The substrate exhibited a strong enhancement factor with LOD as low as 1 × 10^−12^ M, offering a rapid and reproducible response.^93^ Atta *et al.* (2024) in their study titled “Efficient solution-based SERS pesticide analysis using silver–gold nanostars coated with graphene oxide” utilized graphene oxide imprinted with gold–silver nanostars for the detection of organophosphorus insecticides and acaricide in apple. The system offered excellent mechanical stability and an LOD down to 10 × 10^−12^ M.^94^ Bai *et al.* (2024) published a magnetic nanosurface imprinted polymer nanoprobe for dinotefuran detection using Fe_3_O_4_@Au nanosols. This nanosensor achieved efficient magnetic separation and reached a detection limit of 9 × 10^−12^ M.^95^ In 2023, Chen *et al.* reported a novel approach in “Electrostatic assembly of Au@Ag nanorods on filter paper for pesticide detection.” The study showcased a paper-based SERS nanosensor that enabled the detection of non-systemic pesticides on vegetable and fruit surfaces. These nanorods formed dense hotspots on the paper surface, enabling fast and sensitive detection of pesticides on vegetables and fruits.^96^

Tao *et al.* (2023), in their paper “A light-activated fiber-optic SERS nanosensor for thiamethoxam,” developed a highly sensitive fiber-optic SERS nanosensor that utilized electromagnetic enhancement and charge transfer effects to activate the surface. The fiber-optic sensor achieved a LOD of 10^−8^ M for thiamethoxam, proving its viability for remote sensing and miniaturized systems.^97^

Transparent and flexible AuNSs/PDMS-based SERS substrates for *in situ* pesticide residue detection were created by Ma *et al.* (2022). With the Raman signal molecule 4-MBA, the AuNSs/PDMS substrate demonstrated good signal uniformity, sensitivity and stability. The detection concentration of 4-MBA was as low as 10^−8^ M. Methyl parathion (MP) standard solution was then detected using the AuNSs/PDMS substrate. In the range of 4 µg mL^−1^–100 µg mL^−1^, a strong linear connection between MP concentration and SERS intensity at 1342 cm^−1^ was found, with a detection limit of 7.39 × 10^−6^ M. AuNSs/PDMS substrate can be utilized as a technique for quick on-site pesticide inspection of agricultural products since it removes the need for sample pre-processing processes prior to testing.^98^

Pham *et al.* (2022) published a hydrophobic poly(dimethylsiloxane)-redesign silver nanoparticle SERS substrate for detecting pesticides such as imidacloprid, cypermethrin and acephate in mangoes. Their method demonstrated LODs down to 7.82 × 10^−8^ M, offering a practical solution for trace detection in fruit matrices.^98^ Liu *et al.* (2022), in their study “Transparent Au@Ag nanorod arrays for fruit pesticide detection,” applied a self-assembled nanostructured SERS platform for thiamethoxam detection in strawberries, mushrooms and apples. The sensor reached a detection limit of 6.85 × 10^−9^ M.^99^ Furthermore, Bhavya *et al.* (2020) used silver nanocubes as an efficient substrate for detecting thiram. This shape-dependent hotspot formation using Ag nanocubes and nanowires enhanced detection efficiency and signal amplification, resulting in a remarkably low LOD 10^−14^ M.^100^

Tang *et al.* (2019) used a glass bead coated with Ag-NPs as a nonplanar SERS substrate to develop a quick method of detecting pesticide residues. Conventional SERS substrates are typically made as planar devices. The suggested “dip–dry–measure” sensing method avoided the coffee ring, in contrast to the conventional “drop–dry–measure” SERS detection method, which resulted in a high degree of SERS signal homogeneity. Imidacloprid and Chlorpyrifos were detected in the apple extract solution with LOD of 2 × 10^−7^ M and 2.9 × 10^−8^ M, respectively, demonstrating the usefulness of the developed SERS substrate. The suggested non-planar SERS substrate shows promise for real-world use in environmental and food safety monitoring.^101^

Yaseen *et al.* (2019) developed silver-coated gold nanoparticles (Ag@Au NPs) to detect multiple pesticide residues in peach samples. In this study, multi-class pesticide residues like oxamyl (neonicotinoid), profenofos (organophosphate) and thiacloprid (carbamate) in standard solution and peach fruit were simultaneously detected. The Au@Ag NPs, which had a gold core of about 26 nm and a silver layer of roughly 6 nm, produced strong Raman enhancement. The results showed that the SERS approach could accurately determine the characteristic wavenumber of the pesticides (oxamyl, profenofos, and thiacloprid). Additionally, the results showed that the thiacloprid produced in the peach extract had a LOD of 4 × 10^−7^ M and a determination coefficient (*R*^2^) of 0.986. Furthermore, the LOD for oxamyl and profenofos was 2.7–4.2 × 10^−8^ M, with corresponding determination coefficients (*R*^2^) of 0.988 and 0.985. For the detection of oxamyl, profenofos and thiacloprid in peaches, a good recovery (78.6–162.0%) demonstrated the strong SERS activity with improved accuracy.^102^

Yan *et al.* (2019) presented a MIP with incorporated Au-NPs as SERS-based evaluation of prometryn as well as simetryn, two triazine herbicides. Prometryn and simetryn are simultaneously recognized using a class-specific MIP before being determined by a fingerprint signal (at 974 cm^−1^ and 1074 cm^−1^) in the SERS spectra obtained in the presence of AuNPs. The imprinted nanoparticles were used to analyze samples of wheat and rice that had been contaminated with both pesticides. With a LOD of 8 × 10^−8^ M and a relative standard deviation of 1.7–7.8%, the technique offers reasonably acceptable recoveries (72.7–90.9%). Prometryn and simetryn had imprint factors of 5.3 and 4.2, respectively, as compared to non-imprinted polymers (both at 4 × 10^−5^ M of the original solution).^103^

Zhai *et al.* (2017) created prediction models and devised methods for extracting and identifying the Raman signal of combined pesticides. A sensitive and nondestructive method for identifying the combination pesticides acetamiprid, carbendazim as well as chlorpyrifos in apple samples is developed using SERS. The acquired SERS signal intensities of each pesticide in their mixture do not significantly differ from the corresponding pure pesticide's signal intensities at low concentrations, according to the results. The lowest levels of acetamiprid, chlorpyrifos, and carbendazim that can be detected in apples are 2.4 × 10^−8^ M, 1.8 × 10^−7^ M, and 7.3 × 10^−8^ M, respectively. Acetamiprid, chlorpyrifos, and carbendazim have correlation coefficients of 0.893, 0.926, and 0.938, respectively, between projected and actual values.^104^

SERS-based pesticide detection employing nanofinger sensors was reported by Kim *et al.* (2015). Public health needs simple, sensitive, and fast methods to detect even very small amounts of highly toxic pesticides. To address this, they developed a portable sensor system consisting of Raman spectrometer paired with reliable, high-performance gold nanofinger sensor strips. This approach is straightforward, sensitive, rapid and cost-effective compared with standard methods and previously published research that are limited to controlled laboratory settings. The chemical interactions of two pesticides, thiabendazole (TBZ) and chlorpyrifos (CPF) with gold nanofingers were investigated based on the SERS results in order to identify a fingerprint for each pesticide. It was effectively shown that the portable SERS-sensor system could detect TBZ and CPF pesticides in 15 min, with detection limits of 7 ppb on apple skin and 35 ppt in drinking water, respectively.^105^

Silver nanoshells with SERS-active nanostructures for label-free pesticide detection were grown quickly and in a single step, according to Yang *et al.* (2014). Investigated a one-step method for producing Ag nanoshells (Ag NSs) quickly in moderate environments. By transferring a single electron from octylamine to Ag^+^ ions, a homogeneous and full layer of Ag shells was quickly produced on silica core particles in under two minutes at 25 °C without the need for seed metals. The creation of an Ag/EG complex, which significantly increased the reduction potential of the Ag^+^ in ethylene glycol (EG) and made it easier for octylamine to reduce them even at ambient temperature, was thought to be the cause of the exceptionally quick growth of Ag NSs. Thiram on apple peel was successfully identified by the Ag NS-based SERS method down to the level of 38 ng cm^−2^ in a label-free manner, which is highly encouraging for its prospective usage in the on-site detection of residual pesticides.^106^

Lulu *et al.* (2013) created sandwich nanostructures with graphene oxide (GO) incorporated for improved Raman readout and its use in pesticide monitoring. The sandwich nanostructure film has an enhancement factor of approximately 7.0 × 10^−7^ for rhodamine-6G (R6G) and LOD of 0.1 × 10^−6^ M for the pesticide thiram in commercial grape juice. Additionally, the GO embedded sandwich nanostructure can distinguish between different kinds of agricultural chemicals and dithiocarbamate molecules.^107^

To present an extensive review of the practical deployment of SERS-based platforms, Table 3 summarizes a wide range of substrates, target analytes, their respective chemical classes, matrices and detection limits of the analytical approach. These studies collectively demonstrate the progress in designing SERS substrates tailored for various pesticide targets and matrices.

**Table 3:** Representative of recent SERS-based imprinted nanosensors platforms reported between 2010 and 2025 for pesticide detection. The studies are arranged chronologically to illustrate the evolution of substrate design, sensing strategies, and analytical performance

S. no.	SERS substrate	Analyte (target molecules)	Class	Sample/matrix	LODs (M)	Description	Ref.
1	Gold nanoparticles electrodeposited indium tin oxide (AuNPs/ITO) electrode	Acetamiprid	Neonicotinoid insecticide	Vegetable	3.2 × 10^−9^	A MIP-based electrochemical SERS with 13.6 times higher sensitivity compared to SERS	92
2	Graphene-oxide–Ag and graphene-oxide–Ag Ag@ZnO nanocomposites	Thiodicarb	Carbamate	Potato peels	10^−11^ and 10^−12^	Graphene-oxide–Ag and nanocomposites prepared through chemical reduction	93
3	CuO-protected silver nanoparticles modified with β-cyclodextrin	Acetamiprid and pymetrozine	Neonicotinoid and pyridine-azomethine insecticide	Fruits	0.36 × 10^−9^ and 0.94 × 10^−9^	Silver nanoparticles modified by nano-CuO and β-cyclodextrin (β-CD) were created and referred to as β-CD/CuO@AgNPs	108
4	2-Merceptoethylamine (MCE) capped Au@AgNPs	Cypermethrin and lambda-cyhalothrin	Pyrethroid	Pear and apple fruit	1.44 × 10^−8^ and 1.78 × 10^−8^	The surface of the Ag shell optimized Au@AgNP was functionalized with 2-MCE.	109
5	Gold molecularly imprinted polymer solid-state substrates	Fenthion	Organothiophosphate	—	10^−13^	Gold nanoparticle monolayers combined with MIPs	110
6	Gold–silver nanostars coated with graphene oxide	Ziram, phorate, azinphos-methyl and triazophos	Organophosphorus insecticides and acaricide	Apple	10 × 10^−12^, 50 × 10^−12^, 100 × 10^−12^ and 100 × 10^−12^	Inclusion of graphene-oxide with gold–silver nanostars produced several advantages, includes increased sensitivity, colloidal stability, and SERS detection repeatability	94
7	Fe_3_O_4_@MIP nanocatalytic probe-gold nanosol	Dinotefuran	Neonicotinoid	—	9 × 10^−12^	Magnetic nanosurface imprinted polymer nanoprobe	95
8	Terbium metal–organic framework loaded gold nanoparticles (MOFTb@Au)	Malathion	Organophosphate insecticide	—	1.82 × 10^−10^	Surface imprinting technique using MOF_Tb_@Au as the nanosubstrate	111
9	Gold nanosol	Carbendazim	Benzimidazole		8 × 10^−11^	Au@MIP nanosol	112
10	Core–shell AuNPs	Acetamiprid and thiacloprid	Neonicotinoids	Pear and peach samples	1.06 × 10^−7^ and 9.38 × 10^−8^	The imprinted layers at the surface of magnetic nanoparticles were polymerized to create magnetic imprinted polymers	113
11	Optical fiber-based AuNPs SERS sensor	Thiamethoxam	Neonicotinoid	Fruits and vegetables	1 × 10^−8^	High sensitivity with charge-transfer and light-wave excitation	97
12	AgNPs on polydimethylsiloxane (PDMS)	Imidacloprid, and cypermethrin	Neonicotinoid and pyrethroid	Mango	7.82 × 10^−8^, and 1.20 × 10^−7^	Flexible hydrophobic substrate for rapid pesticide detection	98
13	Au@Ag core–shell nanorod arrays	Thiamethoxam	Neonicotinoid	Strawberry, apple, and mushroom	6.85 × 10^−9^	Transparent flexible substrate; good reproducibility	99
14	Fe_3_O_4_@Ag-MIP magnetic substrate	Paraquat	Herbicide	Water samples	3.89 × 10^−10^	MIP-based magnetically retrievable sensor	113
15	Au three-dimensional (3D) nanoparticles	Thiram	Dithiocarbamate	Peach	9.3 × 10^−9^	The 3D structure offers a large enhancement area	114
16	Gold (Au) nanostars	Paraquat	Bipyridylium	Green tea	7.78 × 10^−7^	Star-shaped nanostructures enhance hotspot density	115
17	Silver nanocubes	Thiram	Dithiocarbamate	—	∼10^−15^	Shape-dependent hotspot formation using Ag nanocubes and nanowires enhanced detection efficiency	100
18	AgNPs-embedded cotton swabs	Thiram and thiabendazole	Dithiocarbamate and benzimidazole	Bitter gourds	4.16 × 10^−9^	Swab-based, portable detection	116
19	Snowflake-like gold nanoparticles as SERS substrates	Parathion-methyl, phosmet and triazophos	Organophosphate	Fruit and vegetable peels	1.29 × 10^−10^, 9.88 × 10^−11^, 9.90 × 10^−11^, and 1.01 × 10^−10^	Snowflake-like gold nanoparticles as core–shell SERS probe	117
20	AuNPs-pseudo-paper films	Thiram	Dithiocarbamate	Apple peel	4.57 × 10^−9^	AuNPs are immobilized on a flexible, paper-like substrate, often composed of cellulose or polymer film	118
21	Bipyramid AuNPs	Methyl parathion	Organophosphate	Cucumber	3.75 × 10^−7^	Bipyramidal AuNPs have sharp edges and tips, which serve as excellent hot spots for Raman enhancement	119
				Tomato	1.20 × 10^−7^		
				Apple	1.39 × 10^−7^		
22	AgNPs-coated glass beads	Chlorpyrifos	Organophosphate	Apple	2.85 × 10^−8^	SERS-active glass bead substrates coated with silver nanoparticles are used to enhance the detection sensitivity	101
		Imidacloprid	Neonicotinoid		1.96 × 10^−7^		
23	CNF/AuNPs	Thiram	Dithiocarbamate	Apple juice	2.16 × 10^−7^	A hybrid substrate combining cellulose nanofibers (CNFs) with embedded gold nanoparticles creates a flexible and biodegradable SERS platform	120
24	Au nanoflowers on a glass slide	Carbendazim	Benzimidazole	Red bell pepper	1.31 × 10^−9^	Reproducible SERS enhancement	121
25	Au-nano island film	Thiram	Dithiocarbamate	Apple	2.08 × 10^−8^	Discontinuous gold islands form hotspots	122
26	Klarite™	Dimethoate	Organophosphate	Honey	8.73 × 10^−6^	Commercial substrate for reproducible SERS	123
27	Au-nano finger chips	Thiabendazole	Benzimidazole	Water/apple skin	4.97 × 10^−9^ to 3.48 × 10^−8^	Finger-like protrusions boost enhancement	105
28	Filter paper with multibranched Au-nanoantennas (MGNs)	Methyl parathion	Organophosphate	Apple	1.00 × 10^−7^	Multiple antenna branches form intense hotspots	124
29	AuNPs decorated GMA–EDMA	Disulfoton	Organophosphate	Orange	3.64 × 10^−9^	Polymer-functionalized nanostructures	125
30	Ag dendrites	Thiabendazole	Benzimidazole	Apple	4.97 × 10^−7^	Dendritic structures increase the scattering area	126
31	Optofluidic SERS	Thiram	Dithiocarbamate	Tea-leaf	2.08 × 10^−8^	Optofluidics integrated with SERS	127
32	Au@AgNPs/GO/Au@AgNPs sandwich nanostructure			Grape	1.25 × 10^−7^	A layered sandwich boosts field confinement	107
33	Ag-quantum dots “sponge” nanocomposites	Chlortoluron, atrazine, diuron and tertbuthyl azine	Urea, triazine, phenylurea and triazine	—	9.36 × 10^−7^, 9.27 × 10^−7^, 4.29 × 10^−7^, and 5.03 × 10^−7^	Porous hybrid composite for multiple analytes	128
34	A single cluster of self-assembled Ag NPs	Thiram	Dithiocarbamate	—	1.00 × 10^−7^	Cluster enhances local field strength	129

A comparison of these studies reveals that the analytical performance of MIP-SERS nanosensors is strongly dependent on the interplay between plasmonic substrate design and molecular imprinting strategy. Systems based on Au or Ag nanostructures coated with ultrathin imprinted layers generally exhibit the lowest detection limits because the target molecules are captured in close proximity to electromagnetic hotspots. Magnetic MIP-SERS platforms provide an additional advantage by enabling target enrichment and separation from complex matrices, thereby improving selectivity and practical applicability. Hybrid systems incorporating graphene oxide, metal–organic frameworks, or semiconductor nanomaterials frequently demonstrate enhanced stability, signal repeatability, and resistance to matrix interference. However, these improvements are often accompanied by increased fabrication complexity and higher production costs. Consequently, no single MIP-SERS architecture is universally optimal, and the choice of platform depends on the balance required between sensitivity, selectivity, reproducibility and suitability for real-sample analysis.

### 6.2. Comparison of detection limits and challenges in conventional sensors

MIPs have been widely integrated with different transducer systems to detect pesticide residues. Common detection techniques include electrochemical sensors, colorimetric sensors, fluorescence-based systems and piezoelectric sensors. The following Table 4 summarizes commonly used transducers for pesticide residue detection along with their limitations.

**Table 4:** Overview of different transducers incubated with MIPs for pesticide residue detection

S. no.	Transducer type	Target analyte(s)	Class	LODs (M)	Limitations	References
1	Electro-chemical sensor	Deltamethrin	Pyrethroid	1.53 × 10^−9^	Potential issues with electrode fouling and matrix interference in complex samples	130
		Chlorpyrifos	Organophosphate	1.85 × 10^−11^		131
		Phosmet	Organophosphate	0.010 × 10^−9^		132
		Profenofos	Organophosphate	5.0 × 10^−6^ to 1.0 × 10^−9^		133
		Cypermethrin	Pyrethroid	3.0 × 10^−11^		134
2	Colorimetric sensors	Paraquat	Herbicide	1.0 × 10^−5^	Limited quantitative capabilities and susceptibility to environmental factors like pH and light	135
		Carbaryl	Carbamate	5 × 10^−9^		136
		Carbofuran	Carbamate	1.81 × 10^−8^		137
3	Fluorescence-based sensors	Pirimicarb	Carbamate	7.51 × 10^−6^	Fluorescence quenching and interference from sample matrices can affect accuracy	138
		Carbendazim	Benzimidazole	1.88 × 10^−5^		139
		Imidacloprid	Neonicotinoid	3 × 10^−10^		140
		Organophosphorus compounds	Organophosphate	10^−9^		141
		Carbofuran	Carbamate	3.17 × 10^−8^		142
		Acetamiprid	Neonicotinoid	3.73 × 10^−8^		143
		Acetamiprid	Neonicotinoid	9.88 × 10^−11^		144
		Paraoxon	Organophosphate (metabolite of parathion)	3.63 × 10^−8^		145
		Malathion	Organophosphate	3.03 × 10^−12^		146
4	Piezoelectric sensors	Pirimicarb	Carbamate	2.8 × 10^−11^	High cost and complexity of instrumentation, potential sensitivity issues in liquid phases	147
		Dichlorodiphenyltrichloroethane and hexachorobenzene	Organochlorines	2.12 × 10^−6^ and 2.42 × 10^−6^		148
		Metolcarb	Carbamate	1.15 × 10^−7^	The presence of other interfering compounds such as atrazine, carbaryl, carbofuran and aldicarb was investigated	149
		Pirimicarb	Carbamate	5.0 × 10^−7^		150
		2,4-Dichlorophenoxyacetic acid	Herbicide	2.26 × 10^−8^		151
		Paroxon and carbaryl	Carbamate	5.0 × 10^−8^ and 1.0 × 10^−7^		152

A comparative assessment of these transducers reveals significant differences in detection limits and operational challenges. Electrochemical sensors can detect pesticides at low levels, such as 1.53 × 10^−9^ M for deltamethrin and 1.85 × 10^−11^ M for chlorpyrifos. Still, they are often affected by issues like electrode fouling and interference from other substances. Colorimetric sensors are easy to use and cost-effective but they usually have higher detection limits (1.0 × 10^−5^ M for paraquat) and environmental factors such as pH and light may have an impact on their accuracy. Fluorescence-based sensors offer moderate sensitivity (3.17 × 10^−8^ M for carbofuran, 3.63 × 10^−8^ M for paraoxon) but can be affected by fluorescence quenching and background interference. Piezoelectric sensors are sensitive (5.0 × 10^−7^ M for pirimicarb) but the equipment is expensive and less effective in liquid samples. In comparison, SERS stands out as a highly promising transducer platform. It can achieve extremely low detection limits, often in the picomolar or even femtomolar range, while maintaining high selectivity and stability in complex sample environments.^153^ The integration of nanoparticles—such as gold and silver nanostructures—significantly amplifies the Raman signal by creating localized electromagnetic “hotspots.” These nanomaterials not only enhance signal intensity but also improve substrate uniformity and reproducibility. As a result, the use of nanostructured SERS substrates enables faster and more accurate detection of pesticides and other hazardous analytes.^154,155^ These advantages make SERS a superior alternative for detecting pesticides and other hazardous analytes in environmental and food monitoring applications.

## 7. Challenges and limitations of SERS based imprinted nanosensors in real-world use

The detection of pesticides often demands the identification of extremely low concentrations within complex samples. Sometimes, rapid field detection also becomes necessary because of a sudden release of pollutants. Despite the advantages of SERS in detecting trace-level pollutants, several challenges restrict its widespread application. These challenges include sensitivity constraints, selectivity, stability and difficulties in field deployment. Recent advancements have addressed some of these challenges and broadened the application of SERS in agricultural and environmental monitoring.

### 7.1. Sensitivity and detection limits

**Fig. 5:**
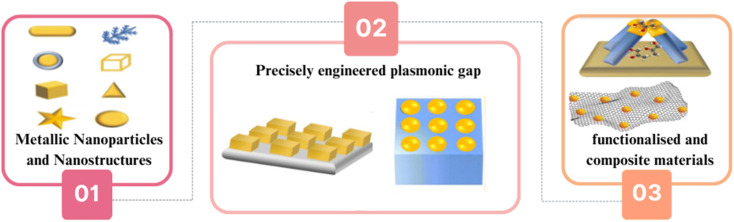
Important structural techniques for increasing the strength of SERS signals: (1) application of various nanostructures and metallic nanoparticles; (2) for the creation of hotspots, precise plasmonic gap engineering; (3) combining composite and enhanced materials to increase stability, repeatability, and selectivity.

Sensitivity is a crucial aspect of SERS as it defines the ability of the nanosensor to detect extremely low concentrations of pollutants. The enhancement in sensitivity largely depends on the properties of SERS substrates, particularly their hot spots and surface area. Sometimes, low concentrations of target analytes do not interact sufficiently with the nanostructured substrate resulting in weak signals. Additionally, interference from background noise and non-specific adsorption can further reduce detection accuracy. To enhance Raman signal amplification and sensor reliability, researchers have focused on optimizing key features of SERS substrates such as nanoparticle shape and composition, controlled plasmonic gap formation and the use of composite or functionalized materials (Fig. 5).^154,155^ In addition to substrate design, coupling SERS with pre-concentration techniques has improved the detection of polar pollutants down to sub-nanomolar levels.^156,157^ Despite these advancements, the effectiveness of SERS varies based on the affinity of target molecules to the substrate.

### 7.2. Selectivity and cross-reactivity

Selectivity is another major challenge in SERS-based detection and becomes even more difficult when dealing with complex environmental and food samples. Many contaminants have similar molecular structures which often causes cross-reactivity and false positive results. To improve selectivity, functionalized SERS substrates incorporating MIPs, antibodies and aptamers have been developed. These enable the selective recognition of target analytes such as pesticides, heavy metals and explosives through specific molecular interactions.^158–160^ Additionally, silver or gold-coated magnetic nanoparticles functionalized with antibodies have demonstrated successful application in SERS-based immunoassays for pathogen detection.^161^ For even higher selectivity in complex samples, researchers have combined SERS with separation techniques like thin-layer chromatography (TLC), capillary chromatography (CC) and capillary electrophoresis (CE). These hybrid approaches make it possible to distinguish multiple analytes effectively and ensure the accurate identification of pollutants.^162,163^ While some investigations include multiple structurally related interferents and validation in real sample matrices, others rely primarily on laboratory-based measurements involving a limited number of competing species. As a result, direct comparison of selectivity performance across different MIP-SERS platforms remains challenging due to the lack of standardized testing protocols and reporting criteria. Future studies should therefore adopt more rigorous selectivity assessments using structurally similar analogues, complex matrices, and quantitative metrics such as selectivity coefficients, imprinting factors, and signal enhancement ratios to enable a more reliable assessment of practical sensor performance.

### 7.3. Stability and reproducibility issues

Reproducibility is a fundamental requirement for any analytical technique to ensure reliability and accuracy. Variations in SERS substrates, such as NPs aggregation, oxidation or degradation over time can result in inconsistent SERS signals and reduced reliability over time. To address this, researchers have developed structured and well-ordered metal NPs as SERS substrates, which provide enhanced reproducibility while maintaining high sensitivity. Advanced nanofabrication techniques, like as optical lithography, vapor deposition and electron beam lithography (EBL) allow for precise control over nanostructure design.^164,165^ Despite these advancements, high-cost and time-consuming fabrication methods remain a limitation for large-scale production. To overcome this, alternative cost-effective strategies such as self-assembly, screen printing and Langmuir–Blodgett (LB) deposition techniques have been explored.^166–168^ These approaches allow for large-area, batch fabrication of SERS substrates with enhanced stability and reproducibility. MIP layers can further improve SERS signal reproducibility by promoting selective localization of target analytes near plasmonic hotspots. In conventional SERS substrates, random adsorption of molecules and non-uniform hotspot distribution often result in significant signal fluctuations. The imprinted cavities within MIPs act as recognition sites that selectively capture target pesticide molecules and position them close to the SERS-active surface, thereby increasing the probability of consistent hotspot interaction. In addition, the polymer shell can protect metallic nanostructures from oxidation, aggregation, and environmental degradation, improving substrate stability during storage and repeated use. Several studies have reported quantitative improvements in reproducibility. For example, Zhao *et al.* (2024) reported a gold nanoparticle monolayer-MIP substrate for fenthion detection with a signal uniformity RSD of 7.05% and a batch-to-batch reproducibility RSD of 10.40%. Similarly, Yan *et al.* observed RSD values of 1.7–7.8% for the simultaneous detection of prometryn and simetryn using AuNP-incorporated MIPs.^103,110^

In addition to substrate engineering, recent studies suggest that advanced calibration strategies can significantly improve the quantitative reliability of SERS measurements. For example, Zhang *et al.* (2023) developed a pseudo-internal intensity reference method that generated robust calibration curves while minimizing the influence of substrate-to-substrate variation and signal fluctuations, enabling reliable quantitative analysis over a wide concentration range.^169^ Similarly, Zhang *et al.* (2024), developed a statistical SERS probability calibration strategy based on the relationship between detection probability and molecular occupancy within SERS hotspots, enabling robust quantification across wide concentration range while minimizing the effects of signal fluctuation and batch-to-batch variability.^170^ The integration of such statistical and machine-learning-assisted calibration approaches with MIP-SERS platforms may provide an effective route toward highly reproducible and quantitative pesticide analysis.

### 7.4. Practical challenges in field applications

For real-world applications such as environmental monitoring and emergency response, portable and field-deployable SERS detection systems are essential. Advances in miniaturized Raman spectrometers and fiber optic-based sensing techniques have significantly improved the feasibility of portable SERS detection. However, traditional colloidal SERS substrates are unsuitable for field applications due to stability issues and difficulties in handling liquid-based samples. In contrast, solid SERS substrates provide a more reliable and portable solution for on-site detection. Techniques such as template-guided self-assembly and bidirectional fiber structuring have been employed to fabricate highly sensitive and reproducible fiber-based nanosensors. These innovations allow for tunable optical responses and controlled “hot spot” densities.^171,172^ These fiber nanosensors are capable of remote sensing and are well-suited for environmental monitoring. However, limited reusability and interference from fiber materials remain challenges. Paper-based SERS nanosensors have also emerged as another promising alternative due to their ease of fabrication, low cost and disposable nature. Different fabrication methods like as dip coating, physical vapor deposition, screen printing and pulse-laser sputtering have further enhanced the practicality of paper-based SERS devices.^173–176^ However, a major challenge with paper-based nanosensors is the potential oxidation of exposed metal NPs, which can degrade SERS activity over time. Beyond fiber and paper-based platforms, the integration of SERS substrates with microfluidic technologies has emerged as another promising strategy for portable and on-site analysis. Microfluidic systems offer several advantages, including controlled sample transport, reduced reagent consumption, improved analytical reproducibility, and compatibility with automated sensing workflows. For example, Zhang *et al.* (2021) developed a highly integrated microfluidic SERS chip capable of detecting Hg^2+^ ions with a detection limit as low as 1 fM, a rapid response within 150 s, and successful validation in real water samples.^177^ Such integrated sensing platforms demonstrate how microfluidics can enhance sample handling and analytical reliability while facilitating field deployment. Similar design principles may be adopted in future MIP-SERS systems to enable rapid, portable, and real-time pesticide monitoring.

## 8. Future perspectives and research directions of SERS based imprinted nanosensors

The development of SERS-based molecularly imprinted nanosensors has shown significant promise in pesticide detection. However, further research is required to address practical limitations and advance their real-world applications. The following points outline key directions for future development:

### 8.1. Enhancing substrate reproducibility and stability

One of the major challenges with SERS nanosensors is the difficulty in obtaining uniform signal intensity from one batch to another. Variations in nanoparticle distribution, hotspot formation and substrate degradation can affect their reliability in routine analysis. To address this, future efforts should focus on engineering ultrathin and uniformly distributed MIP shells that can precisely position analytes within electromagnetic hotspots while simultaneously protecting plasmonic nanostructures from oxidation and degradation. Such designs are expected to further reduce signal variability and improve long-term storage stability of SERS platforms. For example, gold nanodot arrays produced through the electron beam lithography (EBL) have shown highly reproducible SERS performance with controlled hotspot density.^178^ Similarly, techniques such as capillary-assisted assembly and screen printing offer promising and cost-effective methods for batch production of reproducible SERS substrates.^179,180^ To improve long-term stability, methods such as coating AgNPs with inert layers (*e.g.*, Au or silica shells) can prevent oxidation while preserving SERS activity.

### 8.2. Improving target selectivity in complex matrices

Although MIP coatings enhance target selectivity but their binding efficiency in complex food or environmental matrices can still be affected by non-specific adsorption. By optimizing the polymer formulation and using functional monomers that have a high affinity for target pesticides, it is possible to reduce non-specific binding. Additionally, incorporating nanocomposites or dual-recognition systems integrating MIPs with aptamers or antibodies may further increase the selectivity of the nanosensor. As described by Ali *et al.* (2022), such strategies significantly reduce cross-reactivity and improve recognition in mixed samples.^181^

### 8.3. Integration with portable and flexible platforms for on-site testing

**Fig. 6:**
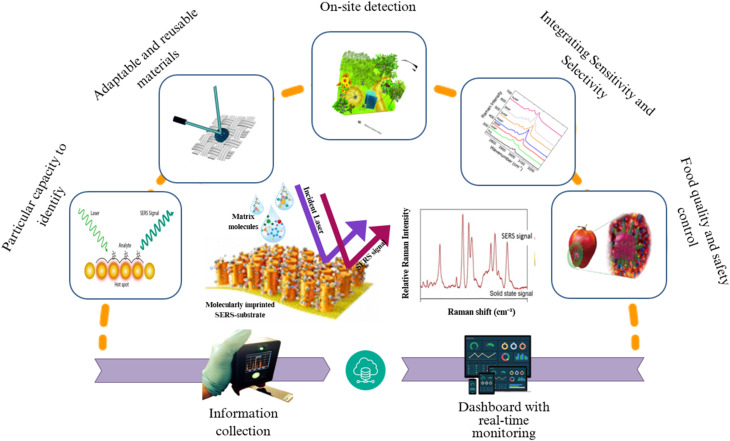
Schematic overview of compact MIP-SERS devices for on-site pesticide detection and identification, highlighting selective recognition, versatile substrates, superior sensitivity and real-time information monitoring.

Despite significant advances in portable and handheld Raman instrumentation, a considerable gap remains between laboratory-scale demonstrations and routine field deployment of MIP-SERS sensors for pesticide monitoring. Many reported platforms achieve excellent analytical performance under controlled experimental conditions; however, practical implementation often requires complex substrate fabrication, sample pretreatment, and carefully optimized measurement conditions (Fig. 6). For example, Logan *et al.* (2022), demonstrated the detection of multiple pesticide residues in basmati rice using handheld SERS coupled with QuEChERS extraction. Although the study highlights the potential of portable SERS for on-site food safety monitoring, sample extraction and matrix-specific optimization were still necessary to obtain reliable analytical performance.^182^ In addition, substrate reproducibility and long-term stability remain important considerations for practical deployment. Variations in nanoparticle morphology, hotspot distribution, molecular imprinting efficiency, and fabrication conditions can lead to batch-to-batch differences in analytical performance. Zhao *et al.* (2024) reported a batch-to-batch reproducibility relative standard deviation (RSD) of 10.40% for a gold nanoparticle monolayer molecularly imprinted substrate used for fenthion detection, indicating that fabrication consistency remains a critical requirement for large-scale applications.^110,183^ Furthermore, plasmonic substrates may undergo oxidation, aggregation, or structural degradation during storage and transportation, potentially affecting sensing performance over time. Although protective coatings such as silica, graphene oxide, polymers, and molecularly imprinted layers have been employed to improve substrate durability and maintain sensing performance. Differences in laser wavelength, detector sensitivity, and spectral resolution can introduce device-to-device variability and affect quantitative prediction models. Moreover, complex food and environmental matrices may contain interfering compounds that influence analyte extraction efficiency, adsorption behavior, and Raman signal intensity, leading to matrix-dependent variations in analytical performance. Therefore, future research should focus on standardized substrate manufacturing, quality-control protocols, long-term stability assessment and validation using real-world samples under field conditions. Addressing these challenges will be crucial for translating MIP-SERS technology from promising laboratory demonstrations into robust and commercially viable platforms for routine pesticide monitoring.

### 8.4. Integration with machine learning for data analysis and multiplex detection

Most SERS-MIP systems are currently designed for single-analyte detection. However, agricultural products are often exposed to multiple pesticides. Developing multiplexed SERS platforms capable of detecting different targets simultaneously would improve monitoring efficiency. Strategies such as incorporating encoding Raman tags or applying machine learning (ML) algorithms for spectral deconvolution can facilitate simultaneous multi-analyte detection. Among the most frequently employed algorithms are principal component analysis (PCA), support vector machines (SVM), support vector regression (SVR), and partial least squares regression (PLSR). PCA is commonly used for dimensionality reduction and extraction of relevant spectral features, whereas SVM-based models facilitate the classification of different pesticide residues and complex sample matrices. For quantitative analysis, regression approaches such as SVR and PLSR have been employed to establish relationships between Raman spectral features and analyte concentrations. These methods improve discrimination capability, reduce the influence of spectral overlap and background interference, and enable the simultaneous analysis of multiple pesticide residues. For example, Jiang *et al.* (2025), demonstrated a SERS platform using Au@Ag core–shell nanostructures for the identification and quantification pf multiple pesticide residues in fruit juices. By combining ML classification and regression (*e.g.*, Support Vector Regression), the system achieved 99.56% accuracy in identifying pesticide types and reliable quantification down to picomolar levels for individual and mixed pesticide residues.^184^ Furthermore, Adhikari *et al.* employed chemometric analysis coupled with SERS for the detection of thiram and carbaryl residues in fruit juices and reported that PLSR models provided the best predictive performance, with superior correlation and lower prediction errors compared with alternative calibration approaches. In another study, a machine-learning-assisted SERS platform using an Ag NPs/NiFeLDH/MXene substrate achieved classification accuracies exceeding 97% through SVM analysis for thiram detection in real fruit-juice samples, further highlighting the utility of ML methods in improving analytical reliability.^185,186^

Despite these promising developments, variations in substrate fabrication, hotspot distribution, laser wavelength, acquisition parameters, and instrument configuration can produce significant spectral variability. In addition, performance is highly dependent on spectral preprocessing procedures, including baseline correction, fluorescence-background removal, denoising, smoothing, and normalization. The lack of standardized preprocessing protocols can result in substantial differences in analytical outcomes and complicate meaningful comparisons between studies. Furthermore, most reported investigations rely predominantly on internal validation or cross-validation, whereas independent external validation using diverse real-world samples and multiple analytical platforms remains limited. Future efforts should therefore focus on establishing standardized spectral-processing workflows and the establishment of large and representative spectral databases to improve the robustness, reproducibility, and practical applicability of ML-assisted SERS technologies for routine pesticide residue monitoring.

### 8.5. Enhancing SERS signal reliability through advanced spectral processing

Beyond conventional enhancements, several advanced SERS methodologies are under development that hold significant promise for next-generation pesticide detection. One such promising direction is UV-SERS, which operates in the ultraviolet region of the spectrum. This approach allows the detection of molecules with UV-resonant absorption, such as aromatic pesticide compounds and biological residues. Although achieving strong enhancements in this region is challenging due to limitations in conventional plasmonic materials like Au and Ag. Researchers have explored alternatives such as Al, Rh and Pt for UV SERS substrates. Notably, enhancement factors of ∼10^2^ have been reported using aluminum-coated substrates at excitation wavelengths of 244–266 nm.^187,188^ Despite lower enhancement factors compared to visible-range SERS, UV-SERS opens up new potential for selective detection of biomolecular targets and pesticide residues in food or plant tissues where UV absorption overlaps with the molecular structure of the pesticide.

Another advancement is the integration of ultrafast and stimulated spectroscopies with high sensitivity of SERS with femtosecond time resolution to monitor dynamic chemical changes. This approach enables real-time monitoring of ultrafast molecular processes such as degradation, hydrolysis or photoactivation on catalytic surfaces.^189^ These systems are valuable for studying reaction kinetics and mechanisms of pesticide breakdown, especially in photocatalytic or light-assisted detoxification environments. Although primarily used in photochemical reaction studies, SE-FSRS can potentially be adapted to track pesticide degradation dynamics or rapid interaction with target molecules, offering both temporal and structural insight at femtosecond resolution. Tip-enhanced Raman spectroscopy (TERS) also presents unique advantages for pesticide detection at the nanoscale. TERS combines atomic force or scanning tunneling microscopy with SERS, producing highly localized electromagnetic enhancement at the metallic tip apex. This enables label-free chemical mapping at the nanometer scale, offering spatial resolution down to 10 nm. In pesticide detection, TERS can be applied to map pesticide residues on plant surfaces, grains or even within plant tissues with subcellular precision.^190,191^ Its application to surface-bound pesticide molecules on fruits or environmental particles could allow for direct and spatially resolved mapping of contaminants. However, challenges such as tip fabrication, stability and reproducibility must be addressed before widespread adoption.

## 9. Conclusions

Compared to conventional analytical techniques, MIP-SERS nanosensors offer the unique advantage of combining ultrasensitive Raman signal amplification with highly selective molecular recognition. The studies reviewed herein demonstrate that the performance of MIP-SERS nanosensors is governed by the synergistic interplay between molecular recognition and plasmonic signal enhancement. Effective sensor design requires not only highly selective imprinted recognition sites but also careful control of nanostructure morphology, hotspot accessibility, and MIP layer thickness to maximize analyte capture and Raman signal amplification. Recent advances have enabled the detection limits to reach down to the femtomolar and picomolar range. For example, silver nanocube-based SERS substrates achieved a limit of detection as low as 10 femtomolar for thiram. Other examples include detection limits of 10^−13^ M for fenthion and triazophos, and 10^−11^ to 10^−9^ M for pesticides such as thiabendazole, carbendazim, phosmet and paraquat. Despite significant progress, several challenges continue to hinder the broader implementation of MIP-SERS platforms for pesticide monitoring. Major bottlenecks include batch-to-batch variability in substrate fabrication, non-uniform hotspot distribution, limited long-term stability assessments and the lack of standardized protocols for performance evaluation and comparison across studies. Furthermore, many reported systems rely on multistep fabrication procedures that may restrict scalability and routine application. Looking forward, future research should focus on improving the field applicability and cost-effectiveness of nanosensor fabrication. Efforts should also be directed toward the development of reusable MIP layers that improve operational sustainability and reduce analytical costs. Moreover, integration with portable Raman instrumentation, microfluidic platforms, and miniaturized sensing devices will facilitate rapid on-site pesticide monitoring. Recent advances in statistical calibration and pseudo-internal referencing strategies also suggest that data-driven calibration models, chemometric analysis, and machine-learning-assisted spectral interpretation could play an important role in improving quantitative reliability and reducing signal variability. Additionally, the development of multiplex sensors that can simultaneously detect multiple pesticide residues will further increase the practical utility of this technology. Collectively, these developments are expected to accelerate the translation of MIP-SERS nanosensors from laboratory-scale demonstrations to robust, field-deployable technologies for food safety, environmental monitoring, and sustainable agricultural management.

## Conflicts of interest

The authors do not have any conflict of interest.

## Data Availability

This review article does not include any original data. All data referenced in the text are publicly available from the cited literature.
